# Seroprevalence of brucellosis in humans and livestock in Sub-Saharan Africa: a systematic review and meta-analysis

**DOI:** 10.1186/s13643-025-03036-2

**Published:** 2025-12-20

**Authors:** David Wagaba, Jacob Mugoya Gizamba, Lawrence Mugisha

**Affiliations:** Makerere University, College of Veterinary Medicine, Animal Resources and Biosecurity, Kampala, Uganda

**Keywords:** Brucellosis, Seroprevalence, Humans, Animals, Sub-Saharan Africa

## Abstract

**Background:**

Brucellosis is a neglected tropical zoonotic disease of public health and economic concern. The disease is maintained within the populations by infected animals, and humans get infected via the consumption of livestock products and contact with post-parturient materials from an infected animal. Understanding the extent and distribution of the disease in both humans and animals is necessary for effective prevention and control in Sub-Saharan Africa (SSA). Therefore, this systematic review and meta-analysis aimed to determine the seroprevalence of brucellosis in humans and domestic livestock in SSA.

**Methods:**

The review protocol was registered with the International Platform of Registered Systematic Review and Meta-analysis Protocols (INPLASY) under registration number: INPLASY2023120123. A comprehensive search was done in six databases: PubMed, OpenAlex, Google Scholar, Semantic Scholar, and Cross-ref, utilizing Medical Subject Headings (MESH). A number of keywords relevant to the subject of study were used to search through the selected online databases for articles published between 01 January 2012 and December 2024. Two hundred forty two full-text articles that fulfilled the eligibility criteria, distributed across 25 countries in SSA, were included in the final analysis. Cross-sectional studies that reported seroprevalence estimates on brucellosis infection for humans and domestic livestock species were included, whereas studies that reported seroprevalence estimates involving wildlife species and outside sub-Saharan Africa were excluded. The random effects meta-analysis model was used to pool the extracted seroprevalence data using R version v4.4.2. Subgroup analysis was performed for species, SSA region, country, diagnostic test, risk of bias, and whether the study was probabilistic or not. Heterogeneity between the studies was represented using *I*^2^ and tau^2^ statistics. A meta-regression model was used to investigate potential sources of heterogeneity. Publication bias was assessed using funnel plots, Peters’ and Egger’s tests, and corrected statistically using trim and fill analysis.

**Results:**

The overall pooled seroprevalence of brucellosis was found to be approximately 5.7% (95% CI 5.01–6.47, tau^2^ = 1.5303, *I*^2^ = 97.6%). The pooled prevalence in humans was 10.15% (95% CI 7.61–13.41), which was higher than all other livestock species except donkeys. Subgroup analysis revealed that the seroprevalence of brucellosis significantly varied extensively between studies among the different species, across SSA countries and regions (East Africa, West Africa, South Africa, and Central Africa), and diagnostic tests. The year of study, species type, and diagnostic test significantly influenced the heterogeneity between studies. There was publication bias according to Egger’s regression test (bias estimate = − 5.8518, *p* < 0.0001). Trim and fill analysis revealed a seroprevalence estimate of 14.14% (95%CI 12.21–16.31) that significantly differed from the observed pooled seroprevalence.

**Conclusion:**

We report high seroprevalence of brucellosis in SSA in both humans and animals based on the current results of the pooled seroprevalence from the studies. The burden is higher in humans compared to domestic livestock; however, this may be underestimated due to fewer studies and challenges with limited diagnostic capacity in most healthcare settings in SSA. The majority of the studies reported in this review utilized mainly RBT and ELISA compared to other sero-diagnostic tests available. Overall, we recommend strengthening biosecurity measures to reduce the burden of brucellosis in humans in all SSA countries as well as improving public awareness of the zoonotic nature of brucellosis to effectively prevent and control brucellosis in both livestock and humans.

**Systematic review registration:**

INPLASY2023120123.

**Supplementary Information:**

The online version contains supplementary material available at 10.1186/s13643-025-03036-2.

## Highlights

• Brucellosis remains a neglected zoonotic bacterial disease that commonly affects regions constrained by limited resources, especially countries in sub-Saharan Africa (SSA).

• The disease burden of brucellosis is very high across SSA and varies greatly with respect to region, country, species, and diagnostic test used.

• The disease burden in the Eastern African region was studied more than in other regions.

• We found a higher disease burden in humans compared to the domestic livestock species, most likely influenced by the nature of studies.

• Limited scope of diagnostic tools being used in the investigational studies for brucellosis despite the recent advances in technologies.

• We found that the Rose Bengal test was popularly utilized to quantify the disease burden by most of the studies.

**Supplementary Information:**

The online version contains supplementary material available at 10.1186/s13643-025-03036-2.

## Introduction

Brucellosis is a significant zoonotic disease of bacterial origin that poses a threat to both animal and public health [1]. It has been described as a re-emerging disease and under the ten(10) top neglected tropical diseases by WHO [25]. Brucellosis is caused by gram-negative, facultative, intracellular coccobacilli that belong to the genus of bacteria,*Brucella.* Six species within the genus *Brucella* are recognized: *B. abortus*, *B. melitensis*, *B. suis*, *B. ovis*, *B. canis*, and *B. neotomae* [3].

The most important Brucella species in animal and public health are *B. melitensis*, *B. abortus*, and *B. suis*, originally known to affect ruminants and pigs, respectively, and yet they are also pathogenic to humans. Among livestock, brucellosis predominantly affects cattle (*B. abortus*), small ruminants, i.e., sheep and goats (*B. melitensis*), and swine (*B. suis*). Brucellosis in sub-Saharan Africa has also been reported to affect other domestic livestock such as donkeys, horses, and camels (*B. abortus* and *B. melitensis*) [12]. Human brucellosis is often non-specific and highly variable with regards to presentation, i.e., patients normally present with undulating fever, headache, chills, myalgia, and arthralgia [14, 25]. It is also known to present with abortion, orchitis, acute renal failure, endocarditis, and encephalitis. The progress of the disease can be acute, subacute, or a chronic relapsing infection [10]. Acquisition of infection in humans has been attributed to contact with infected animals as well as ingestion of fresh and poorly processed animal products. Developed countries exhibit a declining occurrence of brucellosis due to economic stability, critical observation of biosecurity, and improved diagnostic techniques. However, most sub-Saharan countries are low-income and middle-income countries characterized by economic instability and low levels of literacy, culminating in poor observation of biosecurity. Additionally, the lack of sufficient funds and resorting to the use of serological tests, which have lower specificity in endemic areas [23]. Previous studies from sub-Saharan Africa indicate that there is a relatively high burden of neglected zoonotic diseases such as brucellosis. Brucellosis continues to contribute substantially to the burden of non-malarial or non-typhoidal febrile illnesses across sub-Saharan Africa [26]. Additionally, the chronic and relapsing nature of the disease, together with the lack of accessible diagnostic capacity, maintains it as a persistent public health problem. The disease then causes prolonged morbidity, reduced productivity, and economic loss directly affecting the livelihoods of livestock-dependent communities.

This has a potentially negative impact on the economic growth in the areas of livestock production as a result of culling and high abortion rates. However, this burden is often underrepresented as a result of underreporting and few epidemiological studies on brucellosis over time [7, 8].

Therefore, this systematic review and meta-analysis was conducted to determine Brucellosis’s seroprevalence among domestic livestock and humans within sub-Saharan Africa from 2012 to 2024. The results from this study will elucidate areas of focus as well as guide policies in the control of brucellosis in SSA.

## Methods

### Study design

The systematic review and meta-analysis was guided by the updated Preferred Reporting Items for Systematic Reviews and Meta-analyses (PRISMA) guidelines 2020 [22]. The review protocol was registered in the International Platform of Registered Systematic Review and Meta-analysis Protocols (INPLASY) with registration number: INPLASY2023120123 and DOI number: 10.37766/inplasy2023.12.0123.

### Eligibility criteria

The review included peer-reviewed observational cross-sectional studies that reported the seroprevalence of brucellosis among either humans and farm animals/domestic livestock species (i.e., goats, cattle, sheep, pigs, horses, donkeys, and camels) or both carried out within sub-Saharan Africa published between the years January 2012 and December 2024. The inclusion and exclusion criteria are presented below;

### Inclusion criteria

- Participants/study subjects; humans and domestic livestock species.
- Study design: Cross-sectional studies (i.e., whether probabilistic or not) to investigate whether sampling approaches could influence prevalence estimates.
- Outcomes: brucellosis
- Measure of outcome: seroprevalence of brucellosis.
- Publication period: January 2012 to December 2024.
- The papers that clearly defined the diagnostic tests used.

### Exclusion criteria

- Study designs: Experimental studies, qualitative studies, case series, controlled trials, and systematic reviews and meta-analyses.
- Studies conducted outside the sub-Saharan region of Africa.
- Studies that included wild animals were excluded as the main focus of this study is farm animals.

### Search strategy

The search was conducted using Harzing’s Publish or Perish database search software [11] to obtain articles that reported on the seroprevalence of brucellosis among humans and farm animals/domestic livestock species (i.e., goats, cattle, sheep, pigs, horses, donkeys, and camels) within sub-Saharan Africa between January 2012 and December 2024. The databases selected included PubMed, OpenAlex, Google Scholar, Semantic Scholar, and Cross-ref. Medical Subject Headings (MESH) and keywords were used for the search. One by one, the various databases were selected as the search terms were typed into the search box. The following keywords: seroprevalence, prevalence, brucellosis, brucella, humans, animals, livestock, farm animals, goats, cattle, bovine, camels, horses, equine, pig, porcine, donkeys, and sheep were uploaded into the software. These keywords were combined using AND/OR during the database search, PUBMED for instance: (“Seroprevalence” OR seroprevalence OR prevalence) AND (“Brucellosis” OR brucellosis OR Brucella) AND ("Humans OR "Animals" OR livestock OR "farm animals" OR goats OR cattle OR bovine OR camels OR horses OR equine OR pigs OR porcine OR donkeys OR sheep).

### Data management and screening

The identified articles (3003) were uploaded to the Rayyan AI online tool [21] for screening and removal of duplicates. The articles were first screened based on their titles and abstracts by authors DW and LM for eligibility. Title and abstract screening were performed with more attention placed on the country of the study, whether it was an observational study, species involved, study period as well as other previously highlighted eligibility criteria. For those that qualified, the full texts of the articles were retrieved and further screened if they fully satisfied the inclusion criteria verified by author JG. The article screening is summarized as seen in the PRISMA flow diagram.

### Data extraction

The extracted data from the included studies was exported to a Microsoft Excel file and checked for any outstanding errors and inconsistencies, which were thereafter exported to R software for further analysis. For all the included studies that had fulfilled the inclusion criteria, data was extracted based on the following variables;

- Authors’ name/citation and year of publication.
- Study characteristics i.e. Study title, abstracts, country, year of publication, sample size, numbers of positive/seroprevalence reported, diagnostic tests used, risk of bias, and whether the study was probabilistic or not.
- Study subjects: humans and farm animals/domestic livestock species (i.e., goats, cattle, sheep, pigs, horses, donkeys, and camels)

### Risk of bias

The included studies were evaluated for their quality using the appraisal tool for cross-sectional studies, AXIS [6]. It comprised 20 questions that assess the internal and external validity of the studies as well as the response rate within the studies. The questions addressed the sample/sampling appropriateness, ethics, conflicts of interest, response rate reporting, aims, appropriateness of the study population, consistency and description of the results, description of the data and methods, as well as the limitations. We employed a scoring technique to assess the risk of bias for the studies by assigning scores as previously used in other studies [20] where a “yes” response was scored “1 point” and a “no” response “0 points”. Just two questions which stated: “Does the response rate raise concerns about non-response bias?” and “had the author’s interpretation of the results been influenced by any funding sources or conflicts of interest?” were given a reverse score of “− 1 point” for each “yes” answer. Out of 20 points, the quality of the studies was rated, with scores ranging from 1 to 7 for low quality, 8 to 14 for medium level, and 15 to 20 for excellent quality as shown in (Supplementary file 1) [20].

### Publication bias assessment

Publication bias for both human and animal studies in this review was assessed by carrying out a comprehensive review and funnel plots [2, 18]. Additionally, Peters’ regression and Egger’s regression tests for funnel plot asymmetry were also used.

### Data analysis

Data from the extracted studies was analyzed using a meta-analysis {meta}, {dmetar}, and {metafor} packages used in R statistical software version v4.4.2. The random effects model was employed for the meta-analysis to pool the seroprevalence data and obtain the heterogeneity between the studies. Heterogeneity between the studies was assessed using Higgin’s *I*^2^ values and tau^2^ values. Subgroup analyses were performed per region within SSA (i.e., Eastern, Western, Southern, and Central), country, species, diagnostic test, risk of bias, and whether the study was probabilistic or non-probabilistic. Trim and fill analysis was done to obtain the publication bias-corrected estimate. Sensitivity analysis was carried out while considering influence statistics. The pooled seroprevalences together with other statistics were presented in tables and figures. Heterogeneity investigation was carried out using meta regression methods. Variables used in subgroup analysis were considered, as well as potential moderators, and were considered for inclusion when they had *p*-values less than 0.2 for the test of moderators. Moderators with p-values less than 0.05 were considered significant at multivariable analysis.

## Results

### Literature search

**Fig. 1:**
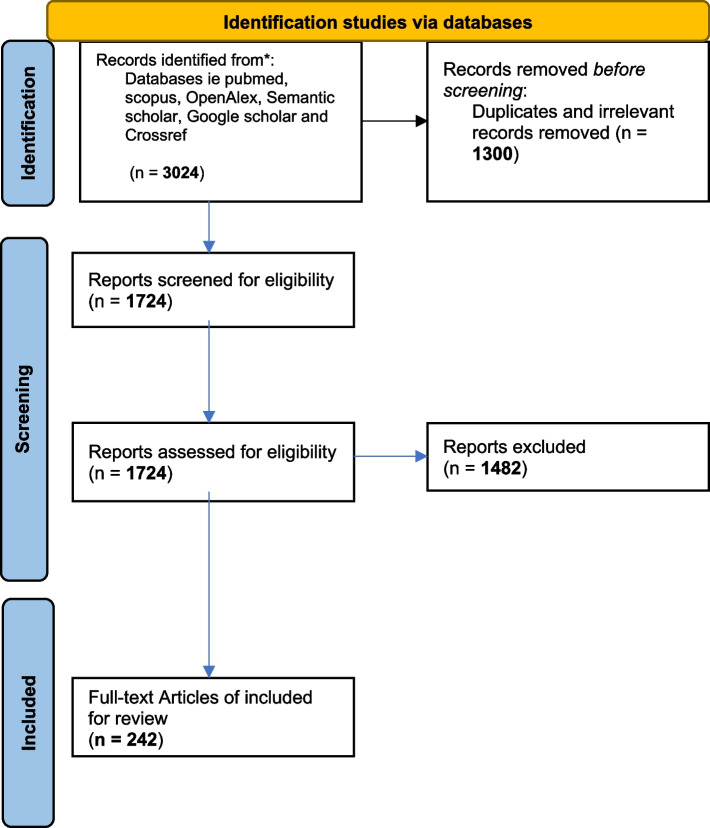
PRISMA flow diagram illustrating article selection process

The database literature search resulted in 3024 records, of which 1724 remained after removing duplicates and other irrelevant articles (*n* = 1300 articles) that were obtained. Of the 1724 articles that remained, both abstract and full-text screening were assessed for eligibility. One thousand four hundred eighty-two articles were excluded because they did not meet the eligibility criteria. 242 full-text articles that met the eligibility criteria were included for the meta-analysis as presented in the PRISMA chart (Fig. 1).

### Overall summary of the studies included

A total of 242 studies were included in this review collectively from 25 countries across SSA. Of these, 17.4% (*n* = 60) studied brucellosis seroprevalence in humans, with only 15.7% (*n* = 38) limited to humans alone. About 75.2% (*n* = 182) studied seroprevalence among domestic animals only. The majority of the studies, i.e., 65.3% (*n* = 158), were of high quality as per the risk of bias assessment. Similarly, most of the studies, i.e., 68.2% (*n* = 165), employed probabilistic methods of sampling.

### Seroprevalence of brucellosis in humans and livestock

From the 242 full-text articles, 344 records were extracted for conducted for the meta-analysis as some study articles considered more than one species. The overall pooled seroprevalence for brucellosis was 5.7% (95% CI 5.01–6.47) with high heterogeneity (tau^2^ = 1.5303, *I*^2^ = 97.6%).

### Subgroup analysis

Subgroup analysis was carried out based on species, risk of bias, whether the study was probabilistic, region, country, and diagnostic test used.

**Fig. 2:**
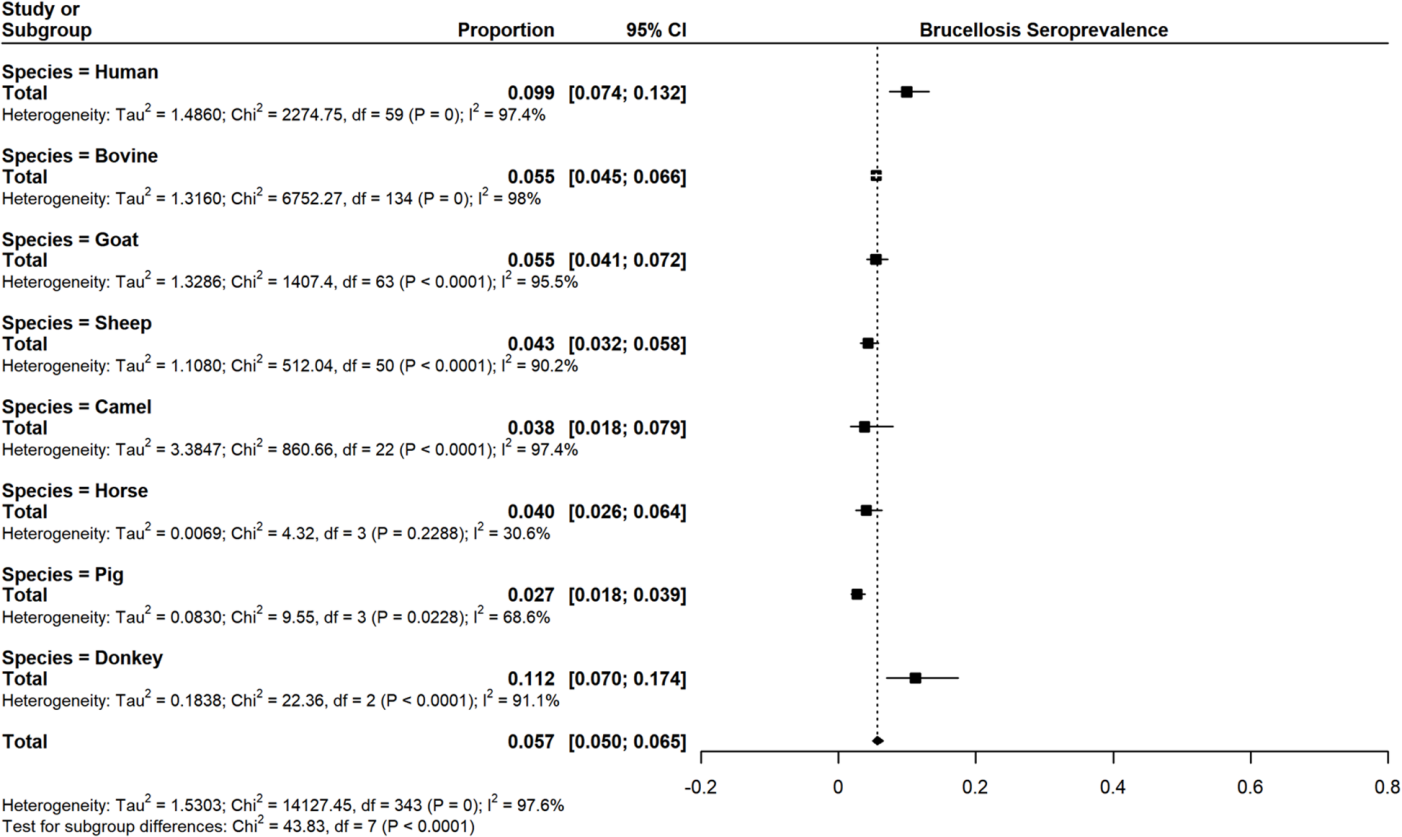
Forest plot showing pooled seroprevalence estimate and sub-grouped by species

Basing on species (Table 1), the highest seroprevalence of brucellosis was recorded among donkeys at 11.2% (95% CI 7.01–17.43), whereas the lowest was among pigs at 2.69% (95% CI 1.85–3.91). Most study records were available for the bovine species compared to all other species. Figure 2 shows the forest plot for the overall estimate sub grouped by species.

**Table 1:** Sub-group analysis by species

Variable	No. of records	Seroprevalence (95% CI)	Tau^2^	*I*^2^
Overall	**344**	5.70 (5.01–6.47)	1.5303	**97.6%**
Bovine	135	5.47 (4.53–6.59)	1.3160	98.0%
Goats	64	5.46 (4.12–7.18)	1.3286	95.5%
Human	60	9.94 (7.44–13.16)	1.4860	97.4%
Sheep	51	4.32 (3.20–5.82)	1.1080	90.2%
Camels	23	3.84 (1.81–7.95)	1.1080	97.4%
Horses	4	4.05 (2.55–6.36)	0.0069	30.6%
Pigs	4	2.69 (1.85–3.91)	0.0830	68.6%
Donkey	3	11.2 (7.01–17.43)	0.1838	91.1%

Considering the risk of bias (Table 2), studies with medium risk exhibited the highest pooled seroprevalence of brucellosis at 6.26% (95% CI 5.12–7.63) and lowest among high-risk studies: 4.28% (95% CI 2.29–7.83). There was no significant difference between the pooled estimates as they had overlapping confidence intervals.

**Table 2:** Sub-group analysis by risk of bias

Variable	No. of records	Seroprevalence (95% CI)	Tau^2^	*I*^2^
Overall	**344**	5.70 (5.01–6.47)	1.5303	**97.6%**
Risk of bias				
Low risk	238	5.48 (4.66–6.45)	1.7137	98.0%
Medium risk	103	6.26 (5.12–7.63)	1.1323	95.2%
High risk	3	4.28 (2.29–7.83)	0.1240	63.9%

Most study records were from studies which conducted based on probabilistic methods of sampling (252/344) and registered a lower pooled brucellosis seroprevalence of 5.48% (95% CI 4.71–6.37) as shown in Table 3.

**Table 3:** Sub-group analysis by whether the study was probabilistic or not

Variable	No. of records	Seroprevalence (95% CI)	Tau^2^	*I*^2^
Overall	**344**	5.70 (5.01–6.47)	1.5303	**97.6%**
Whether study was probabilistic				
Yes	252	5.48 (4.71–6.37)	1.5617	97.7%
No	92	6.33 (4.99–8.01)	1.4332	97.1%

Although the majority of the study records were registered from the eastern region of Africa, southern Africa recorded the highest pooled brucellosis seroprevalence of 10.74% (95% CI 7.51–15.14), while central Africa recorded the lowest: 4.67% (95% CI 2.89–7.46) (Table 4). The higher number of study records in eastern Africa could be explained by a large number from Ethiopia. Despite this, the highest brucellosis seroprevalence by country was from Swaziland at 16.12% (95% CI 14.41–17.99). The lowest seroprevalence was recorded by Eritrea at 1.08% (95% CI 0.77–1.50), as shown in Table 5.

**Table 4:** Sub-group analysis by region

Variable	No. of records	Seroprevalence (95% CI)	Tau^2^	*I*^2^
Overall	**344**	5.70 (5.01–6.47)	1.5303	**97.6%**
Region				
Eastern Africa	259	5.44 (4.64–6.37)	1.7588	97.7%
Western Africa	58	6.20 (4.90–7.83)	0.8717	97.5%
Central Africa	14	4.67 (2.89–7.46)	0.8038	97.3%
Southern Africa	13	10.74 (7.51–15.14)	0.8038	95.1%

**Table 5:** Sub-group analysis by country

Variable	No. of records	Seroprevalence (95% CI)	Tau^2^	*I*^2^
Overall	**344**	5.70 (5.01–6.47)	1.5303	**97.6%**
Country				
Ethiopia	122	3.67 (2.91–4.63)	1.7147	97.1%
Nigeria	40	7.3 (5.60–9.46)	0.7687	97.6%
Tanzania	35	6.07 (3.59–10.06)	2.5749	97.4%
Uganda	29	8.27 (6.10–11.13)	0.7619	98.2%
Kenya	26	9.59 (6.49–13.95)	1.1465	98.3%
Sudan	21	9.97 (6.62–14.75)	0.9842	96.5%
Somalia	11	4.00 (2.32–6.81)	0.7633	80.6%
Cameroon	6	3.46 (1.85–6.39)	0.5813	92.2%
Rwanda	5	9.91 (6.53–14.77)	0.2403	93.8%
Chad	5	4.51 (2.70–7.44)	0.2768	84.9%
Zambia	4	10.94 (7.06–16.58)	0.2209	94.0%
South Sudan	8	13.94 (6.70–26.76)	1.2357	87.9%
South Africa	4	6.83 (2.53–17.17)	1.0726	97.6%
Togo	3	5.02 (1.37–16.75)	1.2852	94.2%
Niger	3	1.72 (0.69–4.20)	0.5695	86.9%
Ivory Coast	3	6.84 (4.24–10.84)	0.1312	85.0%
Guinea	3	3.70 (0.67–17.92)	2.1856	90.9%
Ghana	3	4.79 (2.22–10.06)	0.3614	88.3%
DR Congo	3	9.06 (2.37–29.01)	1.3359	91.8%
Zimbabwe	2	12.94 (8.92–18.41)	0.0824	95.4%
Burkina Faso	2	5.15 (3.64–7.23)	0	0.0%
Angola	2	15.08 (13.44–16.88)	0	0.0%
Swaziland	1	16.12 (14.41–17.99)	–	–
Eritrea	2	1.08 (0.77–1.50)	0	0.0%
Benin	1	8.85 (7.05–11.06)	–	–

Although a variety of diagnostic tests were used in Table 6, the combination of Rose Bengal test and complement fixation test, RBT and CFT, registered the lowest pooled brucellosis seroprevalence, while SAT and ELISA produced the highest.

**Table 6:** Sub-group analysis by diagnostic test used

Variable	No. of records	Seroprevalence (95% CI)	Tau^2^	*I*^2^
Overall	**344**	5.70 (5.01–6.47)	1.5303	**97.6%**
Diagnostic test				
RBT ELISA	103	5.58 (4.50–6.91)	1.2683	97.8%
RBT	76	7.10 (5.83–8.62)	0.7991	96.2%
RBT CFT	71	2.80 (2.00–3.92)	2.0348	96.4%
ELISA	51	6.89 (5.01–9.39)	1.4432	98.5%
RBT SAT	12	9.80 (6.84–13.85)	0.4198	87.9%
CFT	8	4.84 (1.82–12.25)	2.0599	97.5%
SAT	4	9.76 (2.79–28.95)	1.7687	98.5%
RBT CFT ELISA	4	3.07 (1.31–7.02)	0.6902	92.2%
RBT SAT ELISA	4	15.00 (9.65–22.59)	0.2365	92.7%
STAT	2	10.64 (8.06–13.92)	0	0.0%
STAT ELISA	1	8.81 (6.19–12.40)	–	–
SAT ELISA	1	30.80 (25.15–37.09)	–	–
MAT STAT	1	10.00 (6.75–14.58)	–	–
MAT	1	9.00 (5.74–13.83)	–	–
BAT	1	11.90 (7.97–17.41)	–	–

Among all sub-group analyses, substantial heterogeneity was observed.

### Publication bias

**Fig. 3:**
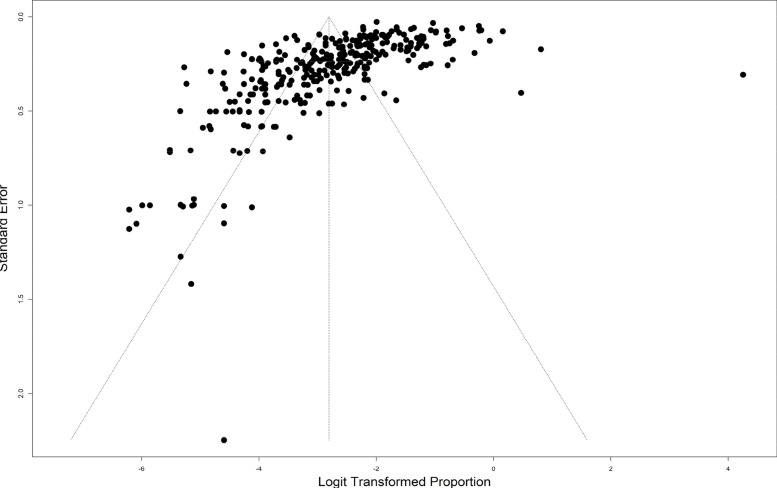
Funnel plot to visualize publication bias (small study effects)

The extent of publication bias in the selected studies was measured and demonstrated by the funnel plot. The funnel plot revealed asymmetry with two outlying study effect sizes (in the right top and left bottom). The Peters’ regression test for funnel plot asymmetry was insignificant (bias estimate = 1.4238, *p* = 0.9531), indicating no significant small study effects. However, the Egger’s test for funnel plot asymmetry was significant (bias estimate = − 5.8518, *p* < 0.0001) (Fig. 3).

### Trim and fill analysis

**Fig. 4:**
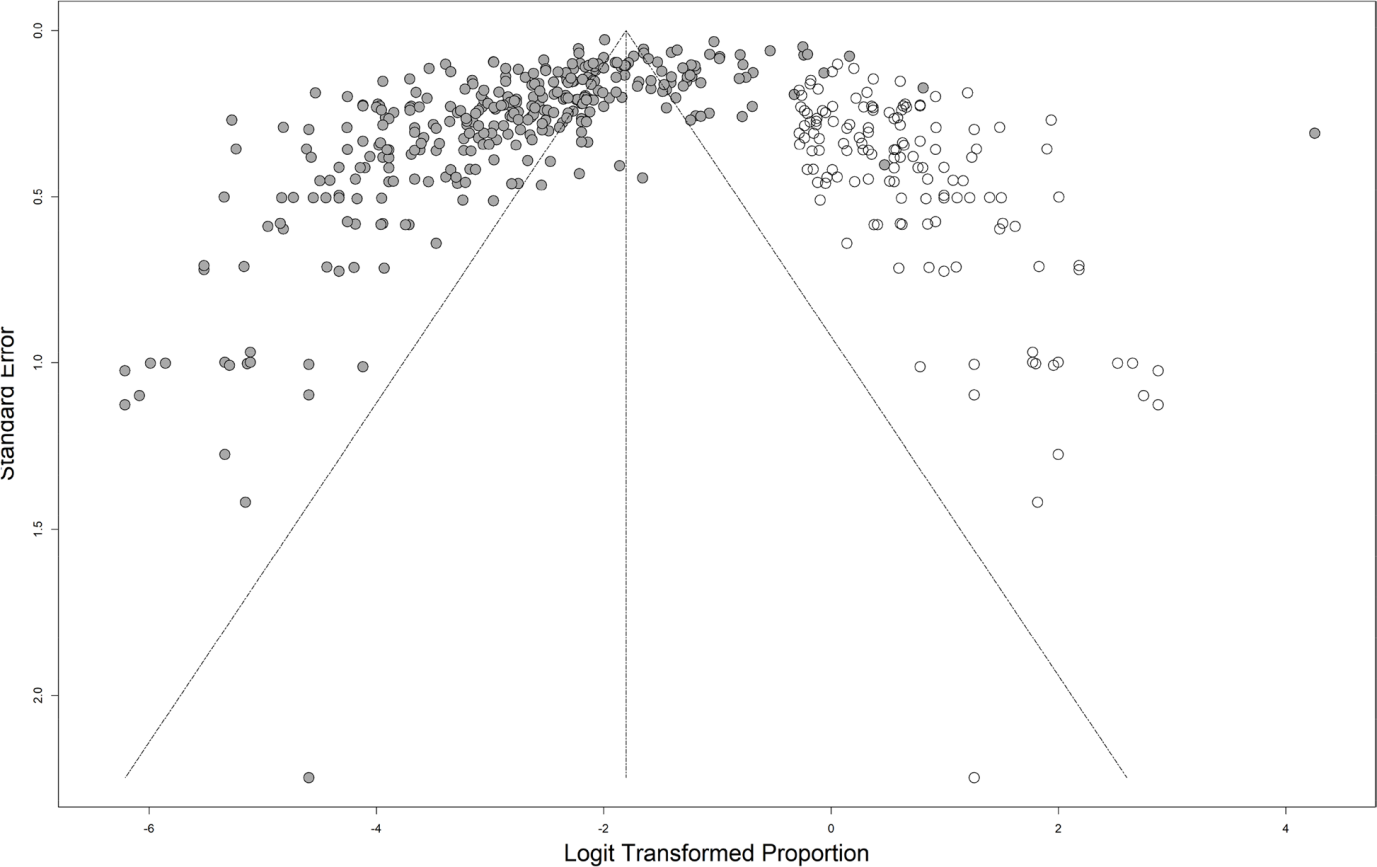
Funnel plot for the trim and fill analysis

Trim and fill analysis was carried out to obtain the bias-corrected overall seroprevalence estimate. The results indicate that the observed pooled seroprevalence is 5.7% (95% CI 5.01–6.47) may be underestimated due to the observed publication bias; 14.14% (95% CI 12.21–16.31) although 142 data points were imputed (indicated by the white circles in Fig. 4). There was still substantial heterogeneity of 97.8% (97.7–97.9). The plot shows that studies with high effect sizes are required to achieve funnel plot symmetry. This is corroborated by the high concentration of points to the left of the plot.

### Sensitivity analysis

Sensitivity analysis was carried out using influence statistics, and studies with high influence were eliminated. Only one study was identified to have high influence on the overall estimate (K. Mekonnen et al., [16]), which reported a seroprevalence of 98.57%. The analysis was rerun to obtain an adjusted pooled seroprevalence estimate. There was no significant change in the seroprevalence estimates, i.e., observed estimate: 5.7% (95% CI 5.01–6.47) versus 5.62% (95% CI 4.97–6.34) after removal of the influential data point.

### Meta regression

This was used to investigate the sources of the observed heterogeneity. The final model shown in Table 7 yielded two variables which accounted for some heterogeneity, i.e., year of study, species, and diagnostic test. Overall, only 17.96% of the original heterogeneity (97.5%) could be explained by the variables.

**Table 7:** Final multiple meta-regression model

Variables	Coefficient (logit)	*P*-value	95% CI
Year of study	**0.043**	**0.033**	0.003; 0.083*
Species			
Bovine	ref	–	–
Goats	− 0.056	0.761	− 0.418; 0.306
Human	**0.443**	**0.024**	0.059; 0.828*
Sheep	− 0.265	0.194	− 0.667; 0.136
Camels	0.076	0.793	− 0.491; 0.642
Horses	− 0.969	0.109	− 2.154; 0.216
Pigs	− 0.644	0.319	− 1.912; 0.624
Donkey	0.547	0.423	− 0.795; 1.888
Diagnostic test used			
RBT ELISA	Ref	–	–
RBT	0.277	0.130	− 0.082; 0.637
RBT CFT	**− 0.566**	**0.005**	− 0.960; − 0.173*
ELISA	**0.422**	**0.034**	0.032; 0.812*
RBT SAT	0.545	0.155	− 0.206; 1.296
CFT	0.045	0.917	− 0.812; 0.901
SAT	0.450	0.455	− 0.734; 1.634
RBT CFT ELISA	− 0.575	0.342	− 1.763; 0.613
RBT SAT ELISA	0.921	0.119	− 0.239; 2.081
STAT	0.288	0.732	− 1.367; 1.943
STAT ELISA	0.207	0.860	− 2.097; 2.512
SAT ELISA	1.520	0.190	− 0.759; 3.799
MAT STAT	0.389	0.741	− 1.928; 2.707
MAT	0.588	0.616	− 1.717; 2.892
BAT	0.370	0.753	− 1.938; 2.679

^*^Significant moderator

## Discussion

This systematic review and meta-analysis were carried out to provide a current synthesis on the seroprevalence of brucellosis among humans and domestic livestock (farm animals) in SSA. Brucellosis continues to be a neglected tropical zoonotic disease in SSA, and this can be reflected by the relatively low recorded number of studies within the study period. This meta-analysis reveals that despite the relatively low number of studies in the region, the presence of brucellosis infection is distributed in all regions within SSA. Generally, the seroprevalence of brucellosis in humans was higher than all the domestic livestock species reported, except donkeys, with a slightly higher pooled seroprevalence. This could be attributed to multiple exposure routes for brucellosis in humans, such as contact, ingestion in milk [10, 17], and the chronic nature of the disease/infection. Similarly, livestock identified with brucellosis on farms are often culled off due to their reduced productivity [4]. In addition to this, humans are more receptive to being tested when there is a high likelihood of infection. This results in the perception of a relatively higher brucellosis burden among humans due to higher reporting of positive cases in humans compared to livestock. There was no significant difference in the seroprevalence estimates when comparing studies which were either probabilistic or not. This reflects that the burden of brucellosis infection is evenly distributed across populations. In a similar perspective, there was no statistically significant difference among the seroprevalence estimates among the categories of risk of bias. The findings could suggest the under-recognition of brucellosis in routine human health surveillance systems, especially with regard to febrile illnesses. This could be explained by high pooled human seroprevalence, which could be attributed to the majority of studies being carried out among individuals of high occupational or environmental exposure. Therefore, this highlights the need to strengthen diagnostic capacity to improve case detection, facilitating subsequent rational antimicrobial use.

Among humans, the overall seroprevalence of brucellosis observed was 10.15% (95% CI 7.61–13.41). Few systematic reviews and meta-analyses have been done summarising the seroprevalence of brucellosis in humans in sub-Saharan Africa. This study is comparable with that of [17] who reported a pooled prevalence of human brucellosis of 16% (95% CI 11–21). This is due to the fact that the study by [17] only investigated occupational exposure to brucellosis. This higher pooled prevalence is expected since only high-risk populations were involved. However, based on the confidence interval of the pooled estimate, there is no significant statistical difference as compared to our study.

There were more studies carried out on the seroprevalence of brucellosis among the domestic livestock species compared to the human studies. The pooled seroprevalences ranged between about 2.69% (95% CI 1.85–3.91) to about 11.2% (95% CI 7.01–17.43) with the lowest among pigs and highest in donkeys. However, this evidence may not be conclusive since studies reporting brucellosis in donkeys were very few. Bovine brucellosis was most popularly researched compared to the other domestic livestock species. This could be due to the fact that cattle have been identified as potential reservoirs of the bacteria for infection in humans [13] either through occupational exposure or contamination. Cattle had higher seroprevalences compared to the other species of livestock with the exception of donkeys which are seldomly researched. This could be attributed to the reporting bias of studies with regard to brucellosis as more economic value has been associated with cattle [1] which attracts more research compared to donkeys. The seroprevalence of brucellosis in goats and sheep was quite similar, i.e., 5.05% (95% CI 3.81–6.66) and 4.05% (95% CI 3.03–5.40) which is also reflected by the fact that they are usually reared together and exhibit similar physiology [19]. This implies that prevention and control strategies suggested for caprine or ovine brucellosis will aid in the reduction of the burden in either species. These findings compare with a review carried out in China [24] where by the identified pooled seroprevalence in sub-Saharan Africa was higher in both species which could be implied by the tropical nature of SSA that allows more of these microorganisms to thrive.

A variety of diagnostic tests were utilized in the determination of the seroprevalence of brucellosis within individual studies included in this review, such as RBT, ELISA, SAT, CFT, STAT, and MAT. The diagnostic tests were either used as a single test or in combinations; however, RBT was popularly reported to be used by most studies irrespective of species. This is probably due to the ease of use and cost-effective nature of the diagnostic test [9]. The pooled seroprevalence of brucellosis identified by studies that reported the use of RBT only was lower compared to other studies that combined it with other tests such as RBT&SAT and RBT&ELISA&SAT; however, it was higher than that in studies where combinations of RBT&ELISA and RBT&CFT were used. This is probably due to the increased sensitivity associated with the former diagnostic test combinations, especially with chronic infections [5, 15]. According to the study by [9] RBT was associated with high sensitivity and specificity, making it a better choice when picking up most exposure-related infections. Combinations with other diagnostic tests, such as ELISA [9], improve the validity of the results, leading to more appropriate representations of the burden within the species. Further research employing the less popular diagnostic tests should be carried out to investigate their reporting of brucellosis burdens in the region.

There was high heterogeneity, i.e., 97.5% between the seroprevalence estimates for the included records. This implies that the studies had highly varying true effect sizes for the seroprevalence of brucellosis. Results from meta-regression analysis revealed that the year of study, species, and diagnostic test used explain this heterogeneity. However, these variables only significantly accounted for about 18% of the observed heterogeneity. It could be stated according to the positive coefficient for year of study that over time, the pooled seroprevalence for brucellosis is slightly increasing. This could be attributed to continuous use of improved diagnostic methods with higher validity. The seroprevalence estimate among humans, while controlling for the year of study and diagnostic test used, was significantly higher compared to cattle, which accounted for part of this high heterogeneity. This corroborates what was stated earlier about the multiple exposure routes for human infection. Additionally, while controlling for species type and year of study, the seroprevalence estimate for studies that utilized RBT&CFT combination and ELISA influenced the overall heterogeneity. This could be due to increased adoption of these diagnostic tests with variations that have higher validity estimates.

With only about 18% of the overall heterogeneity explained by the three variables, a high percentage is left unexplained. This could be due to differences in *Brucella* species infecting the species included in this study. This is the identified study limitation as data concerning *Brucella* species type was not collected. Different *Brucella* species exhibit varying patterns of virulence which could influence their overall infectivity such as *Brucella melitensis*. This should be considered in other meta-analyses to provide a clear picture of the brucellosis burden. Additionally, the unexplained heterogeneity could be due to the study setting of the individual included studies which we recommend for further research. The study setting could also have important variations in climate/weather patterns which would influence the pathogen survival and host–pathogen dynamics. Although this study aimed at describing the current status of brucellosis infection and diagnosis, it brought together studies that used varying diagnostic methods. The most popular diagnostic test, RBT, has been considered as a screening test with high sensitivity leading to a possible increase in the false positive rate. Some studies used it in combination with other tests; however, overall validity estimates were not clearly defined and could also have contributed to the high unexplained heterogeneity. Fewer studies presented from some regions and some with small sample sizes could have produced imprecise pooled seroprevalence estimates. In conclusion, the high unexplained heterogeneity could suggest contextual variability across studies driven by unmeasured factors such as diagnostic thresholds, regional endemicity, and management practices. These factors, together with others, could limit the precision of the pooled estimates, and hence results should be interpreted as indicative rather than definitive.

## Conclusion

This systematic review and meta-analysis were carried out to summarize the seroprevalence of brucellosis in humans and domestic livestock flocks in sub-Saharan Africa between 2012 and 2024. The seroprevalence of brucellosis is widely reported in both humans and livestock species in SSA. Furthermore, the brucellosis seroprevalence was found to be higher in humans, providing evidence of the possibility of zoonotic transmission and the associated risk of the disease in humans. However, the high heterogeneity could imply contextual variability across studies which need to be considered. Public health authorities and clinicians should therefore consider occupational or animal contact as part of the routine diagnosis of brucellosis infection. There is also a need to streamline diagnostic capacity in both research and clinical settings. Further studies are also recommended, aimed at evaluating the causal relationship of the risk factors associated with the spread of brucellosis in humans in order to fully understand the current epidemiology of the disease in SSA. Researchers should also investigate which diagnostic test combinations provide high validity estimates for brucellosis infection status.

## Supplementary Information

Supplementary file 1: A scoring technique to assess the risk of bias for the studies.

## Abbreviations

RBT: Rose Bengal Test
ELISA: Enzyme-linked Immunosorbent Assay
CFT: Complement Fixation Test
SAT: Serum Agglutination Test
STAT: Standard Tube Agglutination Test
MAT: Microagglutination test
BAT: Buffered Brucella Antigen Test
WHO: World Health Organization
SSA: Sub-Saharan Africa
